# Review of Machine Learning Applications Using Retinal Fundus Images

**DOI:** 10.3390/diagnostics12010134

**Published:** 2022-01-06

**Authors:** Yeonwoo Jeong, Yu-Jin Hong, Jae-Ho Han

**Affiliations:** 1 Department of Brain and Cognitive Engineering, Korea University, 145 Anam Rd., Seoul 02841, Korea; boyjeong@korea.ac.kr; 2 Division of Electronics and Display Engineering, Hoseo University, 20 Hoseo-ro79beon-gil, Asan 31066, Korea; yjhong@hoseo.edu; 3 Department of Artificial Intelligence, Korea University, 145 Anam Rd., Seoul 02841, Korea

**Keywords:** deep learning, fundus image, machine learning, retinal image

## Abstract

Automating screening and diagnosis in the medical field saves time and reduces the chances of misdiagnosis while saving on labor and cost for physicians. With the feasibility and development of deep learning methods, machines are now able to interpret complex features in medical data, which leads to rapid advancements in automation. Such efforts have been made in ophthalmology to analyze retinal images and build frameworks based on analysis for the identification of retinopathy and the assessment of its severity. This paper reviews recent state-of-the-art works utilizing the color fundus image taken from one of the imaging modalities used in ophthalmology. Specifically, the deep learning methods of automated screening and diagnosis for diabetic retinopathy (DR), age-related macular degeneration (AMD), and glaucoma are investigated. In addition, the machine learning techniques applied to the retinal vasculature extraction from the fundus image are covered. The challenges in developing these systems are also discussed.

## 1. Introduction

Machine learning methods have been developed and exploited for process automations in many fields and have recently taken a leap forward thanks to the feasibility of deep learning and storage of massive amounts of data. The algorithm is designed to make complex neural networks learn target knowledge from big data. Consequently, traditional machine learning algorithms have been replaced with deep learning methods. As an example, tasks in computer vision have achieved remarkable performance. At the ImageNet Large-Scale Visual Recognition Challenge (ILSVRC) held in 2015, deep learning’s potential was proven by surpassing human performance in recognizing natural images, which is considered a very difficult task [1].

The machine learning applications for images in the medical field include lesion detection, automatic diagnosis, medical image segmentation, and medical image generation. Deep learning was used to classify pulmonary tuberculosis with chest radiographs [2], and a method for detecting and classifying skin lesions in images was proposed [3]. Research was conducted on the diagnosis of Alzheimer’s disease using neuroimaging data [4], and the coordinates of vertebral corners in sagittal X-ray images were predicted for automatic detection [5]. Furthermore, an algorithm was developed for segmenting tumors in multi-modality magnetic resonance images (MRI) of the brain [6]. In addition, multiple sclerosis lesions were also segmented [7]. Part of the knee articular cartilage was extracted from MRI images for the early diagnosis of osteoarthritis [8]. Another clinical application is detecting pulmonary nodules in computed tomography (CT) lung images [9]. For examples of image generation, liver CT images were synthesized and used as the augmented data to train deep learning models to achieve better lesion classification performance [10]. Furthermore, a synthesis method to create brain MRI images was proposed [11], and retinal and neuronal images were generated to enhance data accessibility [12].

Among the various departments in the medical field, ophthalmology is where deep learning applications have been intensely applied. There are two different imaging modalities: fundus imaging and optical coherence tomography (OCT) [13]. Fundus imaging devices use an intricate microscope with an installed image sensor that records the reflected light from the interior surface of the eye. This optical system allows physicians to observe the major biological landmarks inside the eye, as well as the complex background patterns that are created by the inner retinal structures. OCT is a non-invasive imaging modality that incorporates the principles of interferometry and confocal microscopy [14]. With the help of images taken by the two kinds of devices, ophthalmologists can diagnose retinal pathologies and screen patients’ eyes, and those images are the basis for developing ophthalmologic applications.

Many machine learning techniques have been developed in ophthalmology, including applications for the identification of retinal landmarks, retinal pathology segmentation, and retinal disease classification. As a result, reviews of deep learning works have been actively published, with some covering specific domains and some covering ophthalmology in general [15,16,17,18,19,20,21,22,23,24,25,26,27,28,29,30,31,32,33,34,35,36]. A review of the applications in sub-domains has the advantage of providing detailed and rich content, but it can be difficult to note the importance of the applications in a large context, and a review may contain information that is not meaningful from an engineer’s point of view. Conversely, a review of the entire domain may have the merit of exploring commonly used methods, although it may end with a dictionary listing of techniques and without useful information. In this paper, in order to leverage the strengths of both approaches, we restricted our scope to applications of fundus imaging and their most important uses are covered by presenting detailed examples. In particular, we have selected several important, recently published reviews that cover the sub-domain tasks for major eye diseases and the structure of the eye [37,38,39,40]. The following section introduces domain knowledge in the field of ophthalmology, such as the imaging modality, retinal structures, and retinopathy. In the next section, after introducing the general methods for each task, important works are presented in detail. The final section addresses concerns when designing machine learning models and analyzing the results.

## 2. Domain Knowledge

### 2.1. Fundus Image

The fundus image is the reflection of the interior surface of the eye, and it is normally recorded by image sensors, usually in three colors. It includes information about the observable biological structures, such as the surface of the retina, retinal vasculature, the macula, and the optic disc. The spectral range of the blue color enhances the visibility of the anterior retinal layers because the blood vessels and posterior retinal pigment layer absorb it. Meanwhile, the green spectrum is reflected by the retinal pigmentation, providing more information from below the retinal surface, and making its filters can improve the retinal layer visualization. The red spectrum is only related to the choroidal layer beneath the pigmented epithelium, and contains content about the choroidal ruptures, choroidal nevi, choroidal melanomas, and pigmentary disturbances [25].

### 2.2. Retinal Structure

**Figure 1:**
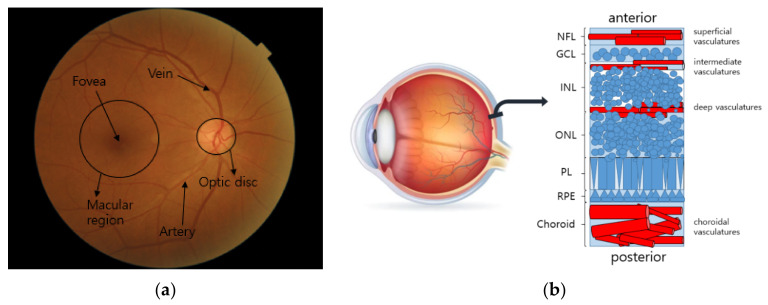
Structure of the fundus: (**a**) retinal landmarks on fundus image; (**b**) structure of seven retinal layers. The NFL, GCL, INL, ONL, PL, and RPE stand for nerve fiber layer, ganglion cell layer, inner nuclear layer, outer nuclear layer, retinal pigment epithelium, and photoreceptor layer, respectively.

The human eye is an organ that takes in visual information in the form of focused light through light-sensitive tissues. When the light reaches photoreceptors that respond to particular spectral regions, the sensed information is converted to electrical signals, and they are transmitted via nerve fibers to the visual cortex of the brain. The signals are then interpreted as visual images in the brain. As depicted in Figure 1a, the eye consists of important biological landmarks. The macula is the central region of the imaging area, where the photoreceptors are highly concentrated for maximum resolving power. The fovea is located in the center of the macular region, and it is responsible for high-acuity central vision. The optic disc, also called the optic nerve head (ONH), is the circular-shaped area on the ocular fundus, and the place where nerve fibers come together to travel to the visual cortex. The retinal vasculature extends from the optic disc and branches out to the retinal layers. As can be seen in Figure 1b, the retinal layers can be divided into seven layers according to the types of cells [41]. The retinal veins can be classified according to their positions. The superficial, intermediate, and deep retinal vessels are located in the nerve fiber layer and along the sides of the inner nuclear layers. The choroidal vessels are located beneath the retinal pigment epithelium (RPE) and Bruch’s membrane. These vessels supply nutrients and oxygen to the photoreceptors [26]. The blood vessels, including the arteries and veins, are well represented in fundus photography with the reflected spectral ranges of red.

### 2.3. Retinopathy

Retinopathy refers to diseases that cause damage to the ocular structures, leading to vision impairment. The three most common retinopathies that affect people around the world have been introduced, and most studies have focused on these retinopathies [20]. They are diabetic retinopathy (DR), age-related macular degeneration (AMD), and glaucoma. DR is a pathology related to abnormal blood flow, and AMD occurs because of the aging of the tissues in the retinal layers. Glaucoma is a group of eye conditions that damage the optic nerve. The detailed descriptions of these diseases are as follows.

#### 2.3.1. DR

Diabetes is a disorder in which not enough insulin is produced or cells are resistant to insulin, which causes blood sugar levels to be abnormally high or low. One of the prevalent consequences of diabetic complications is DR, which causes damage to the overall retinal region. DR can be rated according to an assessment of the lesions represented on the fundus image [32]. The lesions include microaneurysms (MAs), cotton wool spots, hemorrhage, exudate, and neovascularization.

- MA
  It is the most typical lesion and the first visible sign of DR. It is caused by limited oxygen supply. It appears in the form of small saccular structures represented by round red spots with a diameter of 25 to 100 µm [27].
- Cotton wool spot (soft exudate)
  It is an acute sign of vascular insufficiency to an area of the retina found in early DR, and it is also called soft exudate. It appears as white patches on the retina, which is a result of damage to the nerve fibers due to the occlusion of small arterioles, and it causes accumulations of axoplasmic material within the nerve fiber layer [28].
- Hemorrhage
  Retinal hemorrhage refers to bleeding from the blood vessels in the retina caused by high blood pressure or blockage in arterioles. It ranges from the smallest dot to a massive sub-hyaloid hemorrhage. Depending on the size, location, and shape, it provides clues about underlying systemic disorders such as DR and AMD [29].
- Hard exudate
  Hard exudate is caused by increased vascular permeability, leading to the leakage of fluid and lipoprotein into the retina from blood vessels, which are represented as small, sharply demarcated yellow or white, discrete compact groups of patches at the posterior pole [30].
- Neovascularization
  When oxygen shortage occurs in the retinal region due to retinal vessel occlusion, the vascular endothelium grows to overcome the lack of oxygen. This new vessel formation can be extended into the vitreous cavity region and leads to vision impairment [31].

#### 2.3.2. AMD

**Figure 2:**
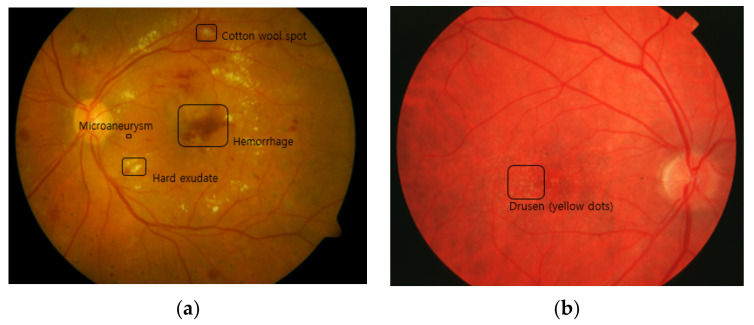
Lesions of the retinopathies: (**a**) MA, exudate, and hemorrhage for DR; (**b**) drusen for AMD.

AMD is a leading cause of blindness in the elderly worldwide. There are two types of AMD: dry AMD and wet AMD. Symptoms of dry AMD are thinning of the macula and the growth of tiny clumps of protein called drusen, which are depicted in Figure 2. Drusen appear over the whole retina as yellow dots in fundus photography, and they are believed to be a result of functional decline due to aging and impaired blood flow—causing problems cleansing waste products from the photoreceptors. Normal individuals over the age of 50 tend to have a small number of drusen, while a large number of drusen are observed in patients with AMD. Wet AMD is caused by newly grown abnormal blood vessels that may leak fluid or blood into the macula causing the macula to lift up from its normally flat position. The neovascularization usually originates in the choroidal vasculature and extends into the sub-retinal space, causing distortion or loss of central vision. AMD can be graded based on these lesions, but there are different schemes for the grading [33,34]. Many machine learning works used AREDS (Age-Related Eye Disease Study) criteria [37]. These criteria divide AMD into eight grades based on the size of the drusen, increased pigment, depigmentation, and geographic atrophy (GA). Wet AMD can be classified based on the presence of neovascularization [33].

#### 2.3.3. Glaucoma

Glaucoma is a common cause of permanent vision loss in which the optic nerve and ganglion cells are damaged. Glaucoma can be diagnosed by examining intra-ocular pressure, optic nerve structure, anterior chamber angle, and the morphology of the retinal vasculature [35]. Glaucoma can be divided into three types depending on how it is caused. The first two types, called open-angle glaucoma and closed-angle glaucoma, are associated with increased intra-ocular pressure. Open angle glaucoma is the most prevalent type of glaucoma. It starts in the anterior part of the eye with a drainage problem. A fluid, called aqueous humor, controls the pressure inside the eye. It is produced behind the iris, and it exits through the trabecular meshwork, which is a mesh-like structure. However, when its exit rate is lower than its production rate, this causes high pressure that damages the optic nerve at the back of the eye. Closed-angle glaucoma happens when the meshwork becomes blocked, resulting in increased pressure. The last type is normal-tension glaucoma. Normal-tension glaucoma occurs without eye pressure. The exact cause of normal-tension glaucoma is still unknown, though it can be diagnosed by observing the optic nerve for signs of damage [36].

## 3. Machine Learning Methods

Machine learning refers to algorithms that can be implemented in systems created for the purpose of solving problems in various fields. It is classified into two main domains by the forms of the target knowledge that must be learned. When information is explicit or directly human-involved, the learning is called supervised learning. Otherwise, learning is called unsupervised or semi-supervised learning, depending on the degree to which the corresponding system independently judges the target-related pattern without human intervention. Supervised learning includes all deep learning methods that exploit labels for training, such as k-nearest neighbor (kNN), a support vector machine (SVM), fuzzy techniques, and random forest (RF), while unsupervised learning includes k-means clustering, fuzzy c-means (FCM), the Gaussian mixture model (GMM), and reinforcement learning.

Another criterion for determining the type of learning is whether the created models include parameters or not. If the data are assumed to have a specific distribution, the model can be set with a fixed number of parameters. Due to prior knowledge, the model has the advantage of a relatively easy-to-understand model. However, there is less flexibility in improving the model with additional data that does not follow the prior distribution. This kind of model includes linear regressions, logistic regressions, Bayesian networks, and artificial neural networks. On the other hand, a non-parametric model made by exploiting the properties of data distribution has the advantage of flexibility and relatively low processing speed. Such models include decision trees, RF, and the k-nearest neighbor (KNN) classifier.

Among the techniques mentioned above, building neural networks for deep learning has recently been considered an efficient way to transform the input data to a condensed representation with end-to-end learning. There are many formats for creating neural networks, depending on how the nodes are interconnected and the basic components of the architecture. A certain number of nodes are grouped together to form a layer, and nodes in the same layer are not usually connected to each other. On the topic of the basic blocks of a neural network, if all nodes in one layer are connected to all nodes of the next layer, the network is called a fully connected network (FCN). If a layer propagates the given information by partially connecting to the nodes of the next layer with regular intervals and the same weights, the network is called a convolutional neural network (CNN). Lastly, if the inference operations proceed by repeatedly feeding the previous outcomes as inputs, the network is called a recurrent neural network (RNN) and is usually designed for handling sequential data.

The methods dealt with in this study are mostly focused on deep learning models, given that deep learning is a state-of-the-art technology that is known to outperform conventional machine learning. However, conventional approaches are also covered for certain tasks if their abilities to analyze necessary patterns and the results are as good as the deep learning methods. This section is divided into two parts. The first part is about the methods for extracting the most complex biological landmark inside the eyes, i.e., retinal vessels. The second part is about the methods that automate the diagnosis and screening of retinal diseases such as DR, AMD, and glaucoma.

### 3.1. Retinal Vessel Extraction Methods

Among the observable landmarks on a fundus image, retinal vessels have the most complex shapes with bifurcations, crossovers, and sudden ends, and the difficulties of the vasculature extraction is proven by the fact that the developed methods so far cannot easily exceed 90% sensitivity, as reported in a recent review [38]. In particular, the deep learning methods do not show good results, and their performances are comparable to or even worse than those of the conventional methods. A brief explanation of the methods used in general for each task is provided, and details for those that have achieved relatively high performance are presented. The public dataset used in those methods and their performances are organized in Table 1 and Table 2, respectively.

The public datasets contain vessel topological information with the segment type or centerline type, as well as the artery and vein marks. The field of view (FOV) and image size differ depending on the dataset. The basic metrics used include sensitivity, specificity, and accuracy. The following are the equations for the counting metrics.

(1) $$\mathrm{Sensitivity}=\frac{\mathrm{Number}\;\mathrm{of}\;\mathrm{true}\;\mathrm{positive}}{\mathrm{Number}\;\mathrm{of}\;\mathrm{true}\;\mathrm{positive}+\mathrm{Number}\;\mathrm{of}\;\mathrm{false}\;\mathrm{positive}}$$

(2) $$\mathrm{Specificity}=\frac{\mathrm{Number}\;\mathrm{of}\;\mathrm{true}\;\mathrm{negative}}{\mathrm{Number}\;\mathrm{of}\;\mathrm{true}\;\mathrm{negative}+\mathrm{Number}\;\mathrm{of}\;\mathrm{false}\;\mathrm{negative}}$$

(3) $$\mathrm{Accuracy}=\frac{\mathrm{Number}\;\mathrm{of}\;\mathrm{true}\;\mathrm{positive}+\mathrm{Number}\;\mathrm{of}\;\mathrm{true}\;\mathrm{negative}}{\mathrm{Number}\;\mathrm{of}\;\mathrm{all}\;\mathrm{predictions}}$$

The true positive and true negative are the predictions of the positive class and the negative class consistent with the ground truth, and the false positive and false negative are the wrong predictions of the positive class and negative class.

The Kappa coefficient is more conservative, thereby eliminating the chance of coincidence.

(4) $$\mathrm{Kappa}\;\mathrm{coefficient}=\frac{p_{o}-p_{e}}{1\;-{\;p}_{e}}{\;\mathrm{where}\;p}_{e}{=p}_{p}{+p}_{n}$$

(5) $$p_{o}=\frac{\mathrm{Number}\;\mathrm{of}\;\mathrm{true}\;\mathrm{positive}+\mathrm{Number}\;\mathrm{of}\;\mathrm{true}\;\mathrm{negative}}{\mathrm{Number}\;\mathrm{of}\;\mathrm{all}\;\mathrm{predictions}}$$

(6) $$p_{p}=\frac{\mathrm{Number}\;\mathrm{of}\;\mathrm{positive}\;\mathrm{predictions}}{\mathrm{Number}\;\mathrm{of}\;\mathrm{all}\;\mathrm{predictions}}\times \frac{\mathrm{Number}\;\mathrm{of}\;\mathrm{positive}\;\mathrm{ground}\;\mathrm{truth}}{\mathrm{Number}\;\mathrm{of}\;\mathrm{all}\;\mathrm{predictions}}$$

(7) $$p_{n}=\frac{\mathrm{Number}\;\mathrm{of}\;\mathrm{negative}\;\mathrm{predictions}}{\mathrm{Number}\;\mathrm{of}\;\mathrm{all}\;\mathrm{predictions}}\times \frac{\mathrm{Number}\;\mathrm{of}\;\mathrm{negative}\;\mathrm{ground}\;\mathrm{truth}}{\mathrm{Number}\;\mathrm{of}\;\mathrm{all}\;\mathrm{predictions}}$$

The p_p_ is the probability that the positive prediction matches the positive ground truth, whereas the p_n_ is the probability that the negative prediction matches the negative ground truth. Accordingly, p_e_ is the probability that the prediction matches the ground truth. The score is then calculated by subtracting the probability of coincidences from the probability of correct predictions, which is then divided by the adjustment term, 1 − p_e_. These metrics can be applied even in the case of multi-classification by extending the binary class to the multi-classes.

#### 3.1.1. Deep Learning Methods for Retinal Vessel Segmentation

**Figure 3:**
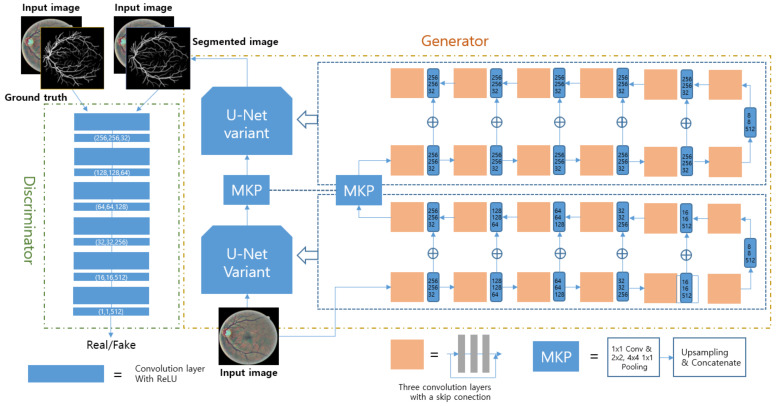
Network architecture design used in [51]. The generator consists of 2 U-Nets, and the discriminator has 6 consecutive convolution layers. The segmented image is input to the discriminator as a pre-processed fundus image. The multi-kernel pooling (MKP) supports the scale invariance of vessel segmentation.

To extract the necessary information from a fundus image, CNN variants were adopted in most of the research on blood segmentation. According to [42,43], RF was used after feature extraction through a CNN. The RF is a non-parametric supervised learner for the final classification of vessels. It was operated after the CNN operation used inputs of the concatenated final features or all the intermediate features that came from the hidden layers of the CNN. Another way to use hierarchical outcomes is to implement an RNN to analyze the global context [44,45,46]. To overcome the shortcomings of CNNs that cannot consider non-local pixel correlations, architectures were modified with skip connection, arithmetic operation, and concatenation [47,48,49,50]. Meanwhile, one widely used deep learning architecture is GAN, which consists of two main parts: generator and discriminator. It is also used for this task, and a recent work achieved good sensitivity with the widely used public datasets DRIVE and STARE [51]. Before feeding the fundus images into the designed network, pre-processing called automatic color equalization (ACE) is performed to enhance the vessels in the image [52]. After that, the image goes into the proposed architecture called M-GAN, as shown in Figure 3. The M-GAN follows the basic shape of the GAN. The generator is made of two consecutive U-Nets that have skip connections with element-wise additions to reflect the global context at each level. The multi-kernel pooling block is located between the two U-Nets to improve the segmentation accuracy. The M-GAN loss function is described in the following equations.

(8) $${\mathrm{generator}\;\mathrm{Loss}=L}_{\mathrm{GAN}}\left(G\right){+L}_{\mathrm{BCE}}{+L}_{\mathrm{FN}}$$

(9) $${\mathrm{discriminator}\;\mathrm{Loss}=L}_{\mathrm{GAN}}\left(D\right)$$

(10) $$\;\;\;\;\;\;\;\;\;\underset{G}{\min}L_{\mathrm{GAN}}\left(G\right)=\frac{1}{2}E_{x}\left[(1\;-\;D{\left(x,\;G\left(x\right))\right)}^{2}\right]\;\;$$

(11) $$\;\;\;\;\;\;\;\;\;\underset{D}{\min}L_{\mathrm{GAN}}\left(D\right)=\frac{1}{2}E_{x,y}\left[\left(1\;-\;D{\left(x,y\right)}^{2}\right)\right]+\frac{1}{2}E_{x}\left[D{\left(x,G\left(x\right)\right)}^{2}\right]\;$$

(12) $$\;\;\;\;\;\;\;\;{\;L}_{\mathrm{BCE}}=-\frac{1}{N}\sum_{i}^{N}\lambda_{\mathrm{BCE}}{(f}_{i}\log\left(f_{i}\right)+\left(1\;-{\;f}_{i}\right)\log\left(1\;-f_{i}\right))\;$$

(13) $$\;\;L_{\mathrm{FN}}=\frac{1}{N_{p}}\sum_{i}^{N}\lambda_{\mathrm{FN}}{\left(1\;-{\;p}_{i}\right)}^{2}{,\;p}_{i}=\left\{\begin{array}{c} {1,\;\mathrm{if}\;p}_{i}\;\geq \;0.5\\ p_{i}{\;\mathrm{if}\;p}_{i}\;<\;0.5 \end{array}\right.$$

For the generator and discriminator network training, the least-squares loss function, as represented in Equations (10) and (11), was used because it is intuitive and effective for mimicking the input distribution [53]. The other two terms, L_BCE_ and L_FN_, were added to the generator loss. L_BCE_ is the pixel-wise entropy function that calculates the differences directly between the ground truth and the segmented outputs, thereby improving performances. L_FN_ is added to reduce the false-negative errors as can be seen in Equation (13).

#### 3.1.2. Other Machine Learning Methods for Retinal Vessel Segmentation

**Figure 4:**
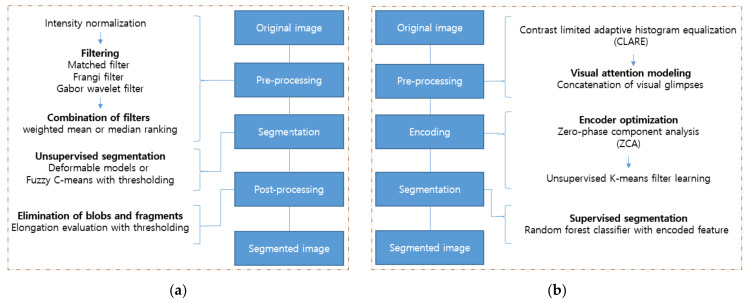
Pipelines of the conventional machine learning methods used for vessel segmentation in [54,55]: (**a**) In [54], three different filters are used for rough segmentation. The deformable models and FCM are used for sophisticated segmentation; (**b**) In [55], combined patches of visual glimpses are encoded with the ZCA and k-means filter. The segmentation is done by the RF classifier with encoded features.

The front part of the other machine learning frameworks generally comprises the image processing techniques, which highlight the information needed—such as using filters. The image processing involves intensity modification and histogram rearrangement, whereas wavelet- and convolution-based filters transform explicit forms in the frequency domain. With condensed representations, the final task is performed by classification algorithms such as SVM, the hidden Markov model (HMM), fuzzy means clustering (FMC), RF, and Adaboost. Within these operations, optimization algorithms can be leveraged to obtain the optimized weights of features or to set the appropriate loss functions, and such a job is done by expectation-maximization (EM), genetic algorithms (GA), and median ranking [38]. Among various works, two pipelines that produce robust results are introduced here [54,55]. The flowcharts of both works are shown in Figure 4.

For the first pipeline, after the intensity normalization, three different filters were applied to enhance the vessel parts of the image: a matched filter, Frangi filter, and Gabor wavelet filter. The matched filter is the modified Gaussian filter, and the Frangi filter is obtained from the eigenvalues of the Hessian matrix. The Garbor wavelet filter uses the Gabor wavelet transform at various scales [56,57,58]. The three filters compensate for each other’s weaknesses, such as noise and the object vanishing problem. After applying the three different filtered images, the combined image is obtained by summing the images and introducing weights. The weights are optimized by the genetic algorithm in a supervised manner [59]. The last phase of this workflow is the classification done by the different techniques, FCM or oriented region-scalable fitting energy (ORSF) [60,61]. FCM separates the vascular pixels from the non-vascular pixels based on a soft threshold, while ORSF makes contours that wrap the vasculature and minimizes the predefined energy that is functional inside and outside of the designated area. The post-process eliminates blobs and objects that are not sufficiently elongated.

As shown in Figure 4, the second pipeline first applies the contrast-limited adaptive histogram equalization (CLAHE) in pre-processing to enhance the contrast between the background and vessels [62]. The second step is to create the visual glimpse patch set by up-sampling and concatenation. Each level of the multi-scale patch set is then utilized to extract the necessary patterns in an unsupervised manner by applying K-means filter learning [63,64]. As represented with Equation (14), the purpose of solving the optimization problem is to obtain a dictionary, $D=\left[d_{1},{\;d}_{2},\ldots ,d_{k}\right]\in R^{p\times K}$, where $p,\;K,{\;d}_{k}$ are the patch size, the number of patterns, and the number of learned centroids, respectively. The harvest through this decomposition is the key that enables local spatial information to be transformed to the encoding domain. Such a process can be done by solving the optimization problem described in Equation (15), where λ is a parameter that controls the reconstruction error and sparsity.

(14) $$<D,\;s>=\underset{D,s}{\mathrm{argmin}}∥\;x\;-{\;\mathrm{Ds}\;∥}_{2}^{2}{\;\mathrm{such}\;\mathrm{that}\;∥s∥}_{0}\;\leq \;1\;,\;s\;\in {\;R}^{K}$$

(15) $$s_{i}=\underset{s}{\mathrm{argmin}}∥x-{\mathrm{Ds}∥}_{2}^{2}+{\lambda ∥s∥}_{1}$$

The RF is then applied as the final task for vessel and non-vessel classification. RF has the advantage of implicit feature selection against overfitting, outliers, and imbalanced data. The RF is made up of 100 decision trees trained independently, with samples drawn with replacement from the encoded training set. The criterion for training is the Gini index, and the RF returns the probability of being a vessel or a non-vessel.

#### 3.1.3. Machine Learning Methods for Retinal Vessel Classification

Though this task has been covered for a short period of time, as proven by the small number of papers relative to the number of papers dealing with blood segmentation, different methods have been used to solve the classification problem [38]. Recent deep learning works exploited CNNs and U-net structures with simple filtering and pre-processing in an end-to-end learning manner [65,66]. The GAN structure was also used with bottom-hat transformed images [67]. On the topic of the traditional classification algorithms used for this research, decision boundary optimization algorithms such as linear discriminant analysis (LDA), SVM, and KNN were used [68,69,70,71]. In addition, RF was applied to leverage its strengths in avoiding overfitting and outliers [72]. The following two works also belong to traditional machine learning and show better performance than deep learning and others in the same category.

**Table 2:** Performances achieved by retinal vessel extraction methods.

Task	Reference	Dataset	Metric (%)		
			Sensitivity	Specificity	Accuracy
Vessel segmentation	[51]	DRIVE	83.46	98.36	97.06
		STARE	83.24	99.38	98.76
	[54]	DRIVE	86.44	95.54	94.63
		STARE	82.54	96.47	95.32
	[55]	DRIVE	86.44	97.67	95.89
		STARE	83.25	97.46	95.02
Vessel classification	[73]	DRIVE	94.2	92.7	93.5
		INSPIRE	96.8	95.7	96.4
		WIDE	96.2	94.2	95.2
	[74]	DRIVE	95.0	91.5	93.2
		INSPIRE	96.9	96.6	96.8
		WIDE	92.3	88.2	90.2

**Figure 5:**
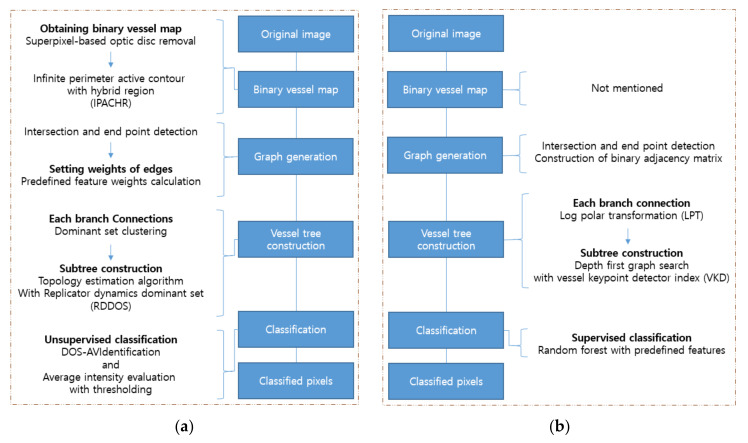
Pipelines of the conventional machine learning methods used for the vessel classification in [73,74]: (**a**) In [73], after the graph generation by the weight calculation with intersection and end point detection, the subtree construction is followed by branch connections. Finally, the classification is done by DOS-AV Identification; (**b**) In [74], the adjacency matrix is constructed during the graph generation, and LPT and VKD are used for vessel tree construction. In the last step, the vessels are classified through the RF with the predefined features.

As shown in Figure 5, the first workflow is divided into two parts: network topology generation by graph representation and clustering based on the pre-defined feature set [73]. In order to construct the vascular network topology, the optic disc is removed and the blood vessels are segmented [75,76]. Next, the segmented vessels are skeletonized with a morphology thinning operation, and bifurcations, crossovers, and blood vessel ends are removed by locating the intersection points and terminal points. Then the neighborhood pixels at the intersections and endpoints form a vertex set, and their edge weights are calculated with the predefined features consisting of the intensities of the three color channels, orientation of the blood vessels, curvature of blood vessels, blood vessel diameters, and entropy. The weight between the two points is defined as the inverse of the Euclidean distance between two feature vectors, and these evaluations are used to construct the weight adjacency matrix for a group of points. The last phase of network topology generation is to make two groups after extracting the dominant sets in each group. For the extraction of the dominant sets, a replicator dynamics dominant set (RDDOS) is used to obtain one dominant set in the given pixel group, and the topology estimation algorithms finds all dominant sets by repeating RDDOS until all elements are assigned [77]. Based on these marked groups, branches that share similarities can be distinguished. Finally, each branch is classified based on the intensity threshold, which is chosen empirically.

The second approach also used graph representation with pseudo labeling [74]. For the first job in graph construction, crossover points and bifurcation points are detected by the vessel key-point detector (VKD) [78]. The VKD is found through the log-polar transform (LPT) of pixels in the set area, and the crossover points and bifurcation points can be distinguished based on the number of angle-span chunks. After that, the neighbor pixels of the chosen points are pseudo-labeled based on their locations and angular spans for each branch connection. By pseudo labeling, the neighbor pixels of all vessel key points are separated into two groups that are not yet declared as artery or vein. With the pseudo marks and VKD, the vessel subtrees are formed through a Depth-First Search (DFS). The final labelling is processed by the RF classifier. Two hundred trees are exploited for the prediction of each vessel subtree. The RF is trained with the pre-defined 66-dimensional feature vector per pixel [79]. A decision for artery labeling is made if more than 90 percent of the vessel pixels that make up the corresponding subtree are classified as artery, and vice versa.

### 3.2. Automation of Diagnosis and Screening Methods

Unlike the extraction of retinal landmarks, building an automated diagnosis and screening system is much more complex work because it should accompany the analysis of the corresponding lesions: exudate, hemorrhage, MA for DR; drusen, depigmentation, and GA for AMD; and glaucomatous optic neuropathy (GON) for glaucoma. In particular, lesions for severe DR and late AMD include neovascularization. Due to these complexities, the deep learning methods have been preferred over the conventional machine learning approaches of setting detailed criteria to satisfy each required feature. For this reason, this section only describes the deep learning methods [37,38,39,40].

Referring to the methods developed so far, the possible ways to develop such systems are by detecting referable cases, detecting the presence of disease, classifying the degree of severity, and localizing lesions. Based on [37,39], the development of frameworks for DR has been the most researched, followed by frameworks for AMD. Frameworks for glaucoma have not been studied as much as DR and AMD [37,39,40]. The DR framework mentioned in this section encompasses not only a general explanation about the developed deep learning methods, but also detailed descriptions of two methods that achieved relatively better sensitivity for the widely used public datasets shown in Table 3 [39]. The same approach is applied to the general explanation in the AMD framework that is mentioned. Furthermore, detailed content about the methods for the classification of multiple AMD stages are presented because multi-classification has been under research in comparison with binary detection, which is a relatively less complicated task. The used dataset with finely-graded labels is presented in Table 3. The last retinal disease to be discussed is glaucoma. Due to the fact that the diagnosis of glaucoma is controversial [80,81,82], the details about a method that adopts the sophisticated procedure of data collection and model creation with glaucoma experts are presented as well as the general approach. The public glaucoma dataset used is listed in Table 3.

The metrics for these tasks include all the metrics in Section 3.1, and the area under the curve (AUC), which indicates the area under the receiver operating characteristic curve (ROC curve). The ROC curve is represented on a two-dimensional plane where the *x*-axis indicates the false positive rate (Equation (17)) or 1-specificity, and the *y*-axis indicates the true positive rate (Equation (16)) or sensitivity. The true positive rate is the proportion of correct positive predictions with respect to the total number of real positive cases, whereas the false positive rate means the proportion of incorrect positive predictions with respect to the total number of real negative cases.

(16) $$\mathrm{True}\;\mathrm{positive}\;\mathrm{rate}=\frac{\mathrm{Number}\;\mathrm{of}\;\mathrm{true}\;\mathrm{positive}}{\mathrm{Number}\;\mathrm{of}\;\mathrm{true}\;\mathrm{positive}+\mathrm{Number}\;\mathrm{of}\;\mathrm{false}\;\mathrm{negative}}$$

(17) $$\mathrm{False}\;\mathrm{positive}\;\mathrm{rate}=\frac{\mathrm{Number}\;\mathrm{of}\;\mathrm{false}\;\mathrm{positive}}{\mathrm{Number}\;\mathrm{of}\;\mathrm{false}\;\mathrm{positive}+\mathrm{Number}\;\mathrm{of}\;\mathrm{true}\;\mathrm{negative}}$$

The ROC curve can be drawn by adjusting the threshold, which slowly increases the false positive rate, or decreases the specificity. The curve usually takes the form that as x increases, y also increases. The AUC takes a value between 0 and 1, and a model having an AUC of 1 is the perfect model for discriminating against any positive case.

#### 3.2.1. DR

As mentioned in Section 2.3.1, DR can be proliferative or non-proliferative depending on the presence of neovascularization, which is newly grown pathologic vessels originating from the existing retinal veins and extending along the inner surface of the retina. Specifically, non-proliferative DR can be divided into several stages according to the severity of the lesions: MA, hemorrhage, and exudates. The possible ways to build automated systems for diagnosis are to make a model only to detect referable DR, or to classify the severity along with the existence of proliferation. If a model can mark the mentioned lesions in the input images, it can be used as a DR screening system. Considerable research has been conducted to develop those systems. For classification, the pre-designed CNN variants have mainly been used [83,84,85,86,87,88,89,90,91]. The pre-trained Inception V3, ResNet152, and Inception-ResNet-V2 were integrated to determine whether DR is referable or not [92], and ResNet34 and VGG16 were exploited to classify non-DR and DR [93,94]. As for multi-classification, the fine-tuned ResNet50, DenseNets, and Inception variants classified four stages of DR [95], while the modified CNN with the pre-trained AlexNet, SqueezeNet, and VGG-16 were also used to distinguish the four stages [96]. Even five-stage divisions were implemented by exploiting the pre-trained VGG, LeNet, AlexNet, and InceptionNet [97,98]. Research for DR lesion detection has been conducted. Exudates were detected by incorporating circular Hough transformation (CHT) followed by classification through a CNN [99]. Another method includes morphological construction and RF [100]. MA and hemorrhage were also detected by patch-based prediction using a customized CNN [101,102]. Two works well represented the automated DR diagnosis and screening system [103,104]. Furthermore, as can be seen from Table 4, they evaluated the performance on the public dataset, which has finer-grained labeling than the other datasets, APTOS 2019 and DDR [39].

**Figure 6:**
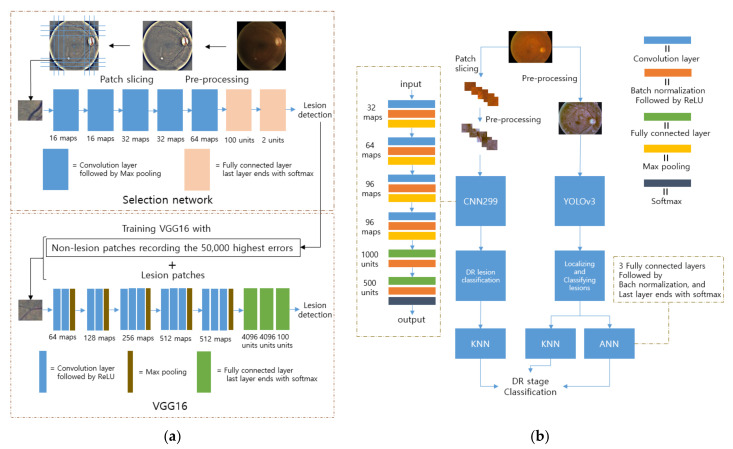
Network architecture design used in [103,104]: (**a**) In [103], the poor classification results from the selection network are used to filter unnecessary data, and the remains are used to fine-tune VGG16, which detects the lesion; (**b**) In [104], various neural networks are followed by KNN or the artificial neural network (ANN) for DR stage classifications. The CNN and YOLO architectures are used for lesion classification, and the YOLO localizes the lesions as well.

The first study is about a screening method for the detection of red lesions such as MA and hemorrhage, which are the first signs of DR [103]. The fundus images are pre-processed using a modified Gaussian filter to enhance the contrast between lesions and non-lesions [102]. After that, the images are divided into same-sized patches to reduce the neural network complexity. Two neural networks are used in total, and the first neural network, called the selection model, consists of five convolution layers with two fully connected layers, as shown in Figure 6a. The selection model infers the probability of lesions. It is built to sample the necessary non-lesion patches to avoid redundant information, given that the number of non-lesion patches is much larger than the number of other patches. After training and inferring all non-lesion patches, 50,000 non-lesion patches that have the highest error are selected. This study also exploited rotated augmented patches and suggested grid pixel sampling, instead of considering all pixels in an image because the goal was to detect the presence of lesions rather than to provide a precise segmentation. The VGG16 was chosen as the inferring model for calculating the probability of the presence of lesions, and pre-trained ImageNet weights were used as the initial weights [84].

The second method classifies five stages of DR and localizes the lesions on the fundus images simultaneously [104]. When it comes to classification, the performances of two separate systems with CNNs and YOLOv3 are compared. The CNNs make decisions based on the features extracted from the input patches, whereas the lesions localized from the whole region of the input images are used as the seed for the decisions by YOLOv3, as depicted in Figure 6b. For the classifier, the KNN is utilized for both models, whereas the ANN is used only with the YOLOv3. Specifically, CNN299 and CNN512 consist of four and six convolution layers with different dimensions of input layers, followed by two fully connected layers. YOLOv3 puts bounding boxes around the objects of interest in the given images and its architecture is well-described in [108]. The ANN classifier has three fully connected layers ending with a softmax layer. As in previous work, transfer learning is also applied with EfficientNetB0 by using the pre-trained weights on the ImageNet dataset only for feature extraction [109]. For dataset utilization, several steps are taken in pre-processing. Contrast-limited adaptive histogram equalization (CLAHE) is used to enhance the contrast, and the Gaussian filter eliminates the noise. Next, after the unnecessary black pixels around the retina are cropped, the color normalization is applied to remove variations that originated from the patients and the cameras. Furthermore, the pre-processed data are augmented with rotation, flipping, shearing, translation, and random darkening/brightening.

#### 3.2.2. AMD

AMD-related deep learning tasks are said to be the second most well-known topic after DR in the ophthalmology domain, according to the number of frequently used datasets and the number of published papers [37,39]. For a brief explanation, AMD can usually be graded based on the areas of lesions, such as drusen, increased pigment, depigmentation, and GA [33,34]. In the binary classification works, no AMD and early-stage AMD images were differentiated from intermediate and advanced-stage AMD images. AlexNet was trained with random initial weights, and its performance was compared with the one with pre-trained weights and the SVM classifier. [110]. The original images and contrast-enhanced images were fed into each ensemble network made of three CNNs with multiple dense blocks and depth-wise separable convolutions. The last colony networks produced the final score by averaging the values calculated by each ensemble network [111]. Another criterion for binary classification is whether the AMD is dry or wet. The three Inception-v3 architectures were exploited for the classification of referable AMD, the assessment of image quality, and the visual availability of the macular region, respectively. Before the given images were fed into the first network, the second and third classifiers first selected gradable images by checking image clarity and capturing the incorrect location of the macular region, thereby preventing the errors that could occur due to poor quality data [112].

For the multi-classification of AMD, three pre-trained overfeat networks were utilized for feature extraction, and areas of interest of various sizes were fed into the networks [113]. The linear SVM was used to classify from two to four stages of AMD, given a concatenated array of the multiple feature vectors [114]. Studies on lesion detection and classification were also conducted. A parallel integration of three Inception-v3 networks was designed for the classification of three stages of drusen, the detection of pigmentary abnormalities, and the detection of neovascularization or central GA [115]. The same principle was applied to differentiate GA from central GA, which is GA around the macular region. Among the three separate models, one was for identifying GA in normal eyes, while the other two were for identifying central GA in normal eyes and differentiating central GA from GA, respectively [116].

**Figure 7:**
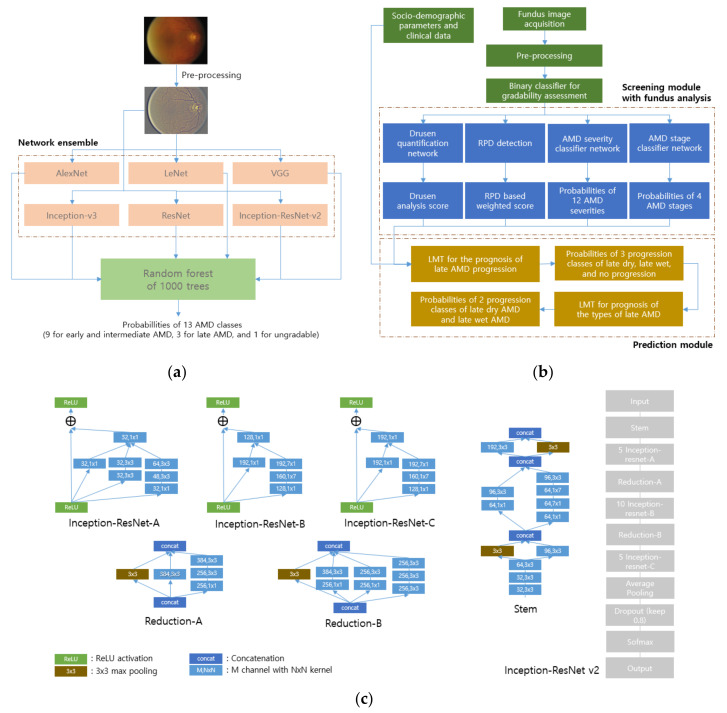
Workflows in [105,106], and one of the used architectures in [105]: (**a**) In [105], an ensemble of 6 different neural networks is used for feature extraction, and the RF is used for the classification of 13 AMD stages; (**b**) In [106], AMD classification as well as fundus analysis with Drusen quantification and RPD detection are conducted in the screening module. Based on the classification, fundus analysis, and additional socio-demographic parameters with clinical data, further prognosis of late AMD is done by the prediction module; (**c**) This shows the architecture of Inception-ResNet v2, one of the six different architectures in [105]. The architecture has six different modules namely, Inception-ResNet-A, Inception-ResNet-B, Inception-ResNet-C, Reduction-A, Reduction-B, and Stem. After analyzing the features by the modules, the Average Pooling and Dropout extract the core information, and the final Softmax layer outputs the probabilities of the classes.

The following works are methods that attempt to distinguish as many stages of AMD as possible. As for the first work, 13 classes were stratified through the network in total [105]. The first stage is defined as few or no AMD-related images and the second to the ninth stages are considered to be the early or intermediate stages. The other three classes are late-stage AMD, wet AMD, and the combined AMD. The last class is for labeling the ungradable images that are unsuitable for grading AMD severity due to overexposure, blurring, or dirt on the lens. The applied pre-processing step normalizes the color and illumination balance by Gaussian filtering. The usual augmentation methods of rotation, mirroring, flipping, cropping, and aspect ratio adjustment are used to regularize the data distribution. For the classification model, six different convolution neural nets were trained, namely ResNet with 101 layers, Inception-ResNet-v2, VGG with 11 convolution layers, Inception-v3, AlexNet, and LeNet [1,83,84,85,86,87,91]. After the six CNNs obtained the class probabilities, the probabilities were fed into an RF with 1000 trees and a final decision was made by a majority vote by the individual trees. The overall workflow is illustrated in Figure 7a.

Other work used a similar pipeline, but several components were added to make sophisticated predictions [106]. Unlike the previous method, the filtering out of ungradable images proceeded with the deep neural networks performing binary classification in the first stage of the system. As shown in Figure 7b, another part of the screening module grades AMD severity in 12 classes, and it consists of 9 classes of early to intermediate AMD and 3 classes of late AMD. Those nine classes are fed into the prediction module created using a logistic model tree (LMT) that predicts whether or not an individual with early or intermediate AMD would progress to late AMD within one or two years. That training is possible because the AREDS dataset that was used has information about the AMD progression of same patients. Furthermore, along with the nine probabilities of severity, other probabilities obtained from the additional two stages—quantifying drusen areas [117] and categorizing reticular pseudo drusen (RPD) into three classes—enter the prediction module. After the first automated prognosis, whether the expected progression is dry or wet AMD is determined at the last stage of the prediction module. This part also consists of two LMT for the one and two-year period, respectively.

The deep learning model used for the binary classification and for four classifications is the ensemble of five networks of different input sizes, including two Inception-v3s, one Inception-ResNet-v2, and two Xception networks. The other models for the 12 AMD classifications and 3 RPD classifications are an ensemble of 6 networks of different input sizes of 1 Inception-Resnet-v2, 2 Inception-v3s, 2 Xception networks, and 1 NasNet [83,85,88,118].

#### 3.2.3. Glaucoma

Glaucoma is a common eye disease, along with DR and AMD. Its symptoms are commonly referred to as glaucomatous optic neuropathy (GON) and includes many features that indicate structural damage, such as thinning or notching of the neuro-retinal rim, retinal nerve fiber layer (RNFL) thinning, disc hemorrhage, parapapillary atrophy (PPA), increased or asymmetric cup-to-disc ratio (CDR), the nasalization of the central optic nerve head (ONH) vessels, the baring of circumlinear vessels, excavation of the optic cup, and laminar dots [119,120]. Although there is no globally agreed-on set of guidelines for glaucoma detection, the features most focused on have been increased CDR, thinning or notching of the neuro-retinal rim, and RNFL defects, which can be reflected in the fundus image [121]. Based on these characteristics, various deep learning methods have been developed and binary classification has been the method of choice in almost all studies [40,122,123]. The Inception-v3 architecture was developed to classify non-referable GON and referable GON. The criteria for manifesting referable GON here is the increased CDR, neuro-retinal rim width, and notches. In addition, the analysis of false-negative classifications by deep learning models was presented to provide the reasons for the wrong decision [124]. The ResNet, a CNN variant, was also used to discriminate glaucoma and used the labels followed by the Japan Glaucoma Society guidelines for glaucoma [125]. In order to evaluate the validity of the neural network, the additional models VGG16, SVM with radial basis function, and random forest were exploited to compare performances [126]. Another work proposed an online learning system with a human–computer interaction loop that used manual confirmation by human graders during training. The adopted architecture was the ResNet and the labels for GON criteria were clearly represented [127]. In addition to the binary classification, methods for multi-classification and feature detection have also been researched. For example, the Inception-v3 network was used with the initial pre-trained weights by ImageNet to output four probabilities corresponding to no-risk, low-risk, high-risk, and likely glaucoma [128]. Furthermore, the network carried out multiple detections of various ONH features for referable GON simultaneously, and ten networks were trained for ensemble prediction by averaging the multiple outcomes [128].

**Figure 8:**
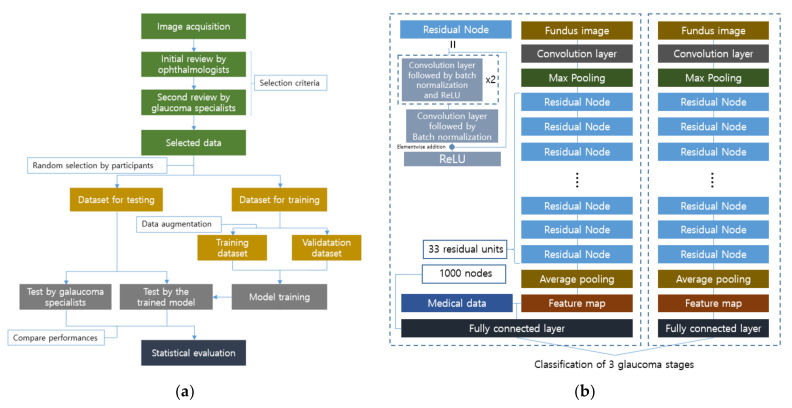
Experimental workflow for the glaucoma classification, and the used architecture in [107]: (**a**) Glaucoma specialists are involved in local data collection and the test of trained neural networks for performance comparison. The significance of the model prediction can be evaluated through the statistical comparison between the experts’ performance and model’s performance; (**b**) The right model obtains a feature map through residual units and pooling layers, and stages are classified by a fully connected layer. The number of classes in the paper was set to three glaucoma stages, i.e., normal eyes, GON-confirmed eyes, and GON-suspected eyes. If necessary, the patients’ medical data can be incorporated into the feature map, and the improvement of the sensitivity and specificity were confirmed according to the paper.

The following method was also developed for multi-classification, and the experimental design is illustrated in Figure 8 and represents the local dataset preparation and utilization well [107]. Two main processes are involved in dataset selection and stratification. They are the assessment of image quality and clinical classification into three categories: normal, GON-suspected, and GON-confirmed. The image quality is controlled by excluding poor images containing severe artifacts, insufficient inclusion of the surroundings of the optic disc or overexposure, and the existence of any retinal pathology other than GON. These image reviews are executed in two consecutive steps by four ophthalmologists and four glaucoma specialists, respectively. After the data is collected, it is divided into two sets for training and testing, with random selection by the participants. The evaluation of the trained model is performed using the test dataset along with two glaucoma experts for comparison.

For the pre-processing of images used for training the model, the Hough transform technique is applied to automatically trim the square region around the ONH center, which is the region of interest for classification [129]. Careful augmentation is done by flipping only horizontally, considering the orientation of the superior and inferior sectors. After that, random cropping and normalization with down-sampling are performed. The model used is the ResNet with 101 residual units, and it is fine-tuned with the pre-trained initial weights from ImageNet to achieve better performance with a smaller amount of data. Furthermore, retrospectively collected medical history data were incorporated into the extracted feature maps to improve the results, as shown in Figure 8. The additional medical data was utilized in a raw format as an array whose elements were normalized, and it contained information about the patients, including the age, personal GON-related therapy, and the personal GON record.

Table 5 illustrates the performance achieved by other methods. In [130], the four severity stages of non-proliferative DR and proliferative DR were classified, and the ungradable class was assigned for the images that cannot be predicted. Furthermore, this work conducted pixel-level annotation with a bounding box that represents the different lesions, such as MA and exudate. The overall accuracy for the six classes were 82.84%. The other method introduced in [131] modified AlexNet to classify DR into four classes including non-proliferative and proliferative. This work used only a green channel because it provides finer details of the optic nerves and other features of the retina, which reduces the distraction for feature analysis from the network’s point of view. The sensitivity and specificity achieved were 92.35% and 97.45%, respectively. For AMD classification, the referable AMD score was calculated by the CNN ensemble and the Image quality was evaluated with the quality score in [111]. The performance of the binary classification was 88.0% accuracy. In [132], this work also detects the AMD with CNN-based architecture that showed 86.3% accuracy. The four works of the automation of glaucoma diagnosis in Table 5 conducted binary classification with the private datasets. In [124,128], Inception-v3 was used for the detection while the other two used ResNet, achieving over 90 percent for the sensitivity and specificity.

## 4. Discussion

According to the investigation, a challenge in the structural component extraction from fundus images is the extraction of the retinal vasculature. Many machine learning methods, including traditional approaches such as graph cuts, matched filtering, morphological image processing, and multi-scale methods, as well as modern approaches like deep learning methods have been developed to improve segmentation performance. Unlike other tasks, such as lesion screening and pathology diagnosis, the vessel extraction still appears to be valid for traditional machine learning techniques, as the performances achieved by some of them have been superior to those of the deep learning methods [38]. This fact shows the limitations of the feature analysis of complicated shapes like blood vessels by deep learning models with a small amount of data, which represents the boundaries of tasks that can be solved more or less effectively with deep learning.

Another thing that can be noted in the application of deep learning to retinal disease screening and diagnosis thus far is that the deep learning models consisting of pre-designed universal models, such as AlexNet, GoogleNet, ResNet, and the Inception network, performed well for binary detection [37,39,40]. However, the developed models were not good at the sophisticated predictions of multiple disease stages. For example, the accuracies of mild DR, moderate DR, and severe DR predictions were 0.2275, 0.6458, and 0.4085, respectively [130], while the accuracy for the binary classification of DR were easily above 0.90 [39]. The accuracy decreased from 99.2% to 96.1% when the AMD detection task was switched to the four stage classification [106]. In another work, the performances of two–four classes of AMD classification were 93.4%, 81.5%, and 79.4%, respectively [114]. Therefore, careful customization of deep learning to improve the performance of stage prediction needs to be researched in a way that interprets the features of each stage independently, rather than the features of all stages at once.

In inferring with deep neural networks, it is important to know which pixels on the given images are critical to the decisions, but the high complexity of the architecture makes it difficult for researchers to analyze the inference process. If researchers can evaluate how much a pixel affects the prediction, researchers can judge whether the designed networks can properly find the region of interest or not. Such efforts have been made in several ways. A primitive way to quantify the importance of pixels is to infer using partially occluded images and compare the performance with the original images. Randomly masked images demonstrated that the neuro-retinal rim region of the fundus images was the most important part, whereas the periphery region was relatively less important for glaucoma detection [107,134]. For AMD stage predictions, a significant drop in confidence was observed when the features of the macular and fovea regions, for their respective class, were masked [105]. Those regions were also recognized as important parts for the kernel visualization techniques with a sliding window [112].

Another visualization technique uses the gradients of the feature maps: class activation map (CAM) and gradient class activation map (Grad-CAM). Basically, they share the same principle of reflecting the weighted importance of each feature map while producing the heatmap. However, CAM provides a stricter basis for the predictions of neural networks than does Grad-CAM because it limits the shape of the network architecture in implementing the global average pooling (GAP) [135]. On the contrary, Grad-CAM has fewer limitations in designing the architecture, while it needs to be supported by propagation methods such as guided backpropagation or layer-wise relevance propagation to improve the precision of the evidence [136]. A method of AMD stage prediction proposed using the heatmap generated by CAM in the last module of the diagnosis framework to distinguish early AMD from late AMD [106]. It showed that the important region was the area around the macula, and the size of the area was different depending on the severity. In a work on glaucoma detection, Grad-CAM was used for visualization, and it proved that the optic disc area was the crucial part [127].

Embedding methods have also indirectly helped elucidate the evidence for decisions. They were originally designed for the purpose of transforming a high-dimensional representation into a low-dimensional representation. Depending on how the transformation criteria are set, many methods have been developed and compared with each other [137]. Among them, t-distributed stochastic neighbor embedding (t-SNE) has a relatively low complexity and is able to preserve local structure well. For these reasons, the reviewed studies adopted it as the method for visualizing the features of the hidden layers. The hidden features for the types of drusen, presence of pigmentary abnormality, and presence of late AMD were represented in each two-dimensional space, and the classification results were justified by presenting evidence of the properly encoded data [115]. Furthermore, one of the vessel segmentation methods showed the relationships between patches of given images in the reconstructed two-dimensional spaces [55]. In spite of the usefulness of an ability to describe the hidden representation, the embedding methods do not explain the reasoning by deep learning frameworks because they are not calculated in a way that links to the inference results.

Leveraging deep learning networks naturally comes with the risk of choosing datasets that have biased distributions. For example, the distribution of collected data may be affected by patient characteristics such as ethnicity, gender, age, and medical history. In addition, the devices used for image acquisition can also be a danger. The fundus images taken from cameras made by different manufacturers are bound to have differences in properties, such as exposure, capture angle, and color distribution [138]. From a data labeling perspective, studies have shown that human grading has limited reproducibility and poor inter-rater reliability [139,140]. Looking at the studies so far, most developed methods were designed to work for only specific diseases, and these disease-specific models cannot make proper decisions when other pathologies coexist in the given images [37,38,39,40]. In summary, researchers should try to avoid erroneous results caused by poor data sources or evaluate the negative impact of the limited data on the results. Such efforts have been made in some works by testing with different test datasets or by analyzing the limitations posed by the dataset conditions. In a DR screening example, the distribution of the area under the ROC curve under a fixed specificity was plotted using six different test datasets [103]. The graphs showed the models’ ability to analyze the respective datasets. If the distribution has a large variance, it can be said that the designed model has a low probability of producing consistent predictions and the model has been trained with relatively diverse data. Although this diverse data cannot be the absolute index, it can provide clues about the relative position on the prior distribution by comparing the respective variances of the metric distributions. One study on glaucoma detection presented the ROC curve generated by several groups in the test data: whole data, non-highly myopic glaucoma patients, and highly myopic glaucoma patients [126]. These validation groups can provide information about model performance for the specific groups of data and biases in the dataset used.

Another thing to consider is overcoming the task-related uncertainty by utilizing additional patient information. The automated diagnosis and screening systems that use information about lesions, which are reflected in the images as simple forms, as the basis for reasoning may be relatively easy to construct compared with systems that handle complex patterns in the images. For example, the shape, size, and color of DR and AMD lesions in fundus images can be more easily distinguished from the background than glaucoma lesions. As can be seen in Figure 2, lesions such as exudate, MA, hemorrhage, drusen, and neovascularization have relatively distinctive properties, and this clarity can provide a solid basis for setting a standard for the severity of diseases [32,33]. However, diseases such as glaucoma have a large number of symptoms, including symptoms that can be detected in fundus images and symptoms that cannot be [119,120]. One related study pointed out that the trained model cannot be a replacement for a comprehensive eye examination for visual acuity, refraction, slit-lamp examination, and eye pressure measurements, because it does not interpret the non-GON lesions [107]. In addition, some of these symptoms do indicate the presence of disease, and no single agreed-on set of guidelines exists [80,81]. Therefore, in order to address the uncertainties, additional ONH features related to the GON assessment were presented to better understand the model’s decision and offer a way to support the final diagnosis by physicians in [128]. This work demonstrated the patient proportions of the RNFL defect presence, disc hemorrhage presence, laminar dot presence, beta PPA, and vertical CDR, with respect to the referral and non-referral cases. Another approach is to use other structural factors as labels rather than using binary assessment by humans. One of the indices for quantification is the RNFL thickness, which is measured by the spectral-domain OCT, and this index has a high reproducibility of measurement and a high correlation with glaucomatous damage [82,141]. The study proved that the encoded features of the fundus image can be mapped to the corresponding RNFL thickness, showing a Pearson correlation coefficient of 0.832 with a *p*-value less than 0.001 [142]. In another case, the minimum rim width relative to the Bruch membrane opening (BMO-MRW) global and sector values were exploited, as they are considered to be a parameter for the indication of visual field loss in glaucoma [143]. It was shown that deep learning predictions were highly correlated with the actual values of global BMO-MRW, with a Pearson correlation of 0.88 and a *p*-value less than 0.001 [144]. This work is expected to support glaucoma diagnosis in the case where the optic discs of highly myopic patients are shown to be difficult to grade [143]. The heatmap generated by CAM confirmed that the neuroretinal rim was a crucial area for the inference.

## 5. Conclusions

This paper presented a review of recent machine learning applications using fundus images in the field of ophthalmology. For automated fundus analysis, methods of retinal vasculature extraction using conventional machine learning techniques as well as the deep learning methods were investigated. Despite the recent abuse of deep learning, traditional approaches have shown its feasibility for this task and it is expected to be helpful in the screening and diagnosis of microvascular diseases of the eye. Furthermore, various deep learning frameworks for DR, AMD, and glaucoma were explored by presenting the characteristics of each disease. In particular, the difficulties of glaucoma diagnosis and the process of glaucoma data collection were described. Finally, this review discussed the limitations and concerns in creating applications and datasets, such as visualization of the basis for the decisions made by deep neural networks, the risk of biased medical data, and patient information utilization to improve performance.

## Figures and Tables

**Table 1:** Public datasets used for the retinal vessel extraction methods.

Dataset	No. Images	Image Size	FOV	Camera	Ground Truth
DRIVE	40	564 × 584	45°	Canon CR6-45NM	A/V label
STARE	50	605 × 700	35°	Topcon TRV-50	Topology and A/V label
INSPIRE	40	2392 × 2048	30°	Carl Zeiss Meditec	A/V label
WIDE	30	1440 × 900	45°	Optos 200Tx	Topology and A/V label

**Table 3:** Public datasets used for automated diagnosis and screening methods.

Diabetic Retinopathy (DR)														
Dataset	Total	Grade												
		Normal		Mild DR			Moderate DR			Severe DR			Proliferative DR	
DIARETDB1	89	27		7			28						27	
IDRiD	516	168		348										
DDR	13,673	6266		630			4713						913	
Messidor	1200	540		660										
APTOS 2019	3662	1805		370			990			193			295	
**Age-Related Macular Degeneration (AMD)**														
Dataset	Total	Grade												
		1	2	3	4	5	6	7	8	9	10	11	12	UG
AREDS	120,656	41,770	12,133	5070	8985	6012	7953	6916	6634	2539	4128	13,260	1098	4158
**Glaucoma**														
Dataset	Total	Grade												
		Normal		Early			Moderate			Deep			Ocular hypertension	
RIM-ONE	169	118		12			14			14			11	

**Table 4:** Performances achieved for automated diagnosis and screening methods.

Retinopathy	Reference	Task	Dataset	Metrics (%)				
				AUC	Accuracy	Sensitivity	Specificity	Kappa Coefficient
DR	[103]	Binary classification for non-severe and severe DR	DIARETDB0	78.6		82.1	50	
			IDRiD	81.8		84.1	50	
			Messidor	91.2		94.0	50	
		Binary classification for DR detection	IDRiD	79.6		85.9	50	
			Messidor	93.6		97.6	50	
	[104]	5 DR stage classification	DDR	97.0	89.0	89.0	97.3	
		5 DR stage classification	APTOS2019	97.3	84.1	84.1	96.0	
AMD	[105]	4 AMD stage classification	AREDS		96.1			
		13 AMD stage classification			63.3			55.47
	[106]	Binary classification		99	99.2	98.9	99.5	
Glaucoma	[107]	3 glaucoma stage classification	RIM-ONE		95.4	96.5	94.1	92.7

**Table 5:** Performances achieved by other methods.

Retinopathy	Reference	Task	Dataset	Metrics (%)			
				AUC	Accuracy	Sensitivity	Specificity
DR	[130]	6 class classification	DDR		82.84		
	[132]	4 class classification	Messidor		96.35	92.35	97.45
AMD	[111]	binary classification	AREDS	94.9	88.0		
	[123]	binary classification	Optretina	93.6	86.3		
Glaucoma	[124]	binary classification	Private dataset	98.6		95.6	92.0
	[126]	binary classification	Private dataset	96.5			
	[128]	binary classification	Private dataset	94.5			
	[133]	binary classification	Private dataset	99.6		96.2	97.7

## Data Availability

The introduced public datasets are available: DRIVE dataset at https://drive.grand-challenge.org (accessed on 1 August 2021); STARE dataset at https://cecas.clemson.edu/~ahoover/stare (accessed on 1 August 2021); INSPIRE dataset at https://medicine.uiowa.edu/eye/inspire-datasets (accessed on 1 August 2021); DIARETDB1 dataset at https://www.it.lut.fi/project/imageret/diaretdb1 (accessed on 1 August 2021); IDRiD dataset at https://ieee-dataport.org/open-access/indian-diabetic-retinopathy-image-dataset-idrid (accessed on 1 August 2021); DDR dataset at https://github.com/nkicsl/DDR-dataset (accessed on 1 August 2021); Messidor dataset at https://www.adcis.net/en/third-party/messidor (accessed on 1 August 2021); APTOS 2019 dataset at https://www.kaggle.com/c/aptos2019-blindness-detection (accessed on 1 August 2021); AREDS dataset at https://www.ncbi.nlm.nih.gov/projects/gap/cgi-bin/study.cgi?study_id=phs000001.v3.p1 (accessed on 1 August 2021); RIM-ONE dataset at https://www.idiap.ch/software/bob/docs/bob/bob.db.rimoner3/stable/index.html (accessed on 1 August 2021).

## References

[1] He K., Zhang X., Ren S., Sun J. Deep Residual Learning for Image Recognition. Proceedings of the 2016 IEEE Conference on Computer Vision and Pattern Recognition (CVPR).

[2] Lakhani P., Sundaram B. (2017). Deep Learning at Chest Radiography: Automated Classification of Pulmonary Tuberculosis by Using Convolutional Neural Networks. Radiology.

[3] Lundervold A.S., Lundervold A. (2019). An overview of deep learning in medical imaging focusing on MRI. Z. Med. Phys..

[4] Jo T., Nho K., Saykin A.J. (2019). Deep Learning in Alzheimer’s Disease: Diagnostic Classification and Prognostic Using Neuroimaging Data. Front. Aging Neurosci..

[5] Cina A., Bassani T., Panico M., Luca A., Masharawi Y., Brayda-Bruno M., Galbusera F. (2021). 2-step deep learning model for landmarks localization in spine radiographs. Sci. Rep..

[6] Ranjbarzadeh R., Kasgari A.B., Ghoushchi S.J., Anari S., Naseri M., Bendechache M. (2021). Brain tumor segmentation based on deep learning and an attention mechanism using MRI multi-modalities brain images. Sci. Rep..

[7] Zeng C., Gu L., Liu Z., Zhao S. (2020). Review of Deep Learning Approaches for the Segmentation of Multiple Sclerosis Lesions on Brain MRI. Front. Aging Neurosci..

[8] Ebrahimkhani S., Jaward M.H., Cicuttini F.M., Dharmaratne A., Wang Y., Herrera A.G.S. (2020). A review on segmentation of knee articular cartilage: From conventional methods towards deep learning. Artif. Intell. Med..

[9] Cui S., Ming S., Lin Y., Chen F., Shen Q., Li H., Chen G., Gong X., Wang H. (2020). Development and clinical application of deep learning model for lung nodules screening on CT images. Sci. Rep..

[10] Frid-Adar M., Diamant I., Klang E., Amitai M., Goldberger J., Greenspan H. (2018). GAN-based synthetic medical image augmentation for increased CNN performance in liver lesion classification. Neurocomputing.

[11] Dar S.U., Yurt M., Karacan L., Erdem A., Erdem E., Çukur T. (2019). Image Synthesis in Multi-Contrast MRI with Conditional Generative Adversarial Networks. IEEE Trans. Med. Imaging.

[12] Zhao H., Li H., Maurer-Stroh S.M., Cheng L. (2018). Synthesizing retinal and neuronal images with generative adversarial nets. Med. Image Anal..

[13] Ilginis T., Clarke J., Patel P.J. (2014). Ophthalmic imaging. Br. Med. Bull..

[14] Aumann S., Donner S., Fischer J., Müller F., Bille J.F. (2019). Optical Coherence Tomography (OCT): Principle and Technical Realization. High Resolution Imaging in Microscopy and Ophthalmology.

[15] Chu A., Squirrell D., Phillips A.M., Veghefi E. (2020). Essentials of a Robust Deep Learning System for Diabetic Retinopathy Screening: A Systematic Literature Review. J. Ophthalmol..

[16] Jiang A., Huang Z., Qui B., Meng X., You Y., Liu X., Liu G., Zhou C., Yang K., Maier A. (2020). Comparative study of deep learning models for optical coherence tomography angiography. Biomed. Opt. Express.

[17] O’Byrne C., Abbas A., Korot E., Keane P.A. (2021). Automated deep learning in ophthalmology: AI that can build AI. Curr. Opin. Ophthalmol..

[18] Charry O.J.P., Gonzales F.A. (2020). A systematic Review of Deep Learning Methods Applied to Ocular Images. Cienc. Ing. Neogranad..

[19] Pekala M., Joshi N., Liu T.Y.A., Bressler N.M., Debuc D.C., Burlina P. OCT Segmentation via Deep Learning: A Review of Recent Work. Proceedings of the Asian Conference on Computer Vision (ACCV).

[20] Li T., Bo W., Hu C., Kang H., Liu H., Wang K., Fu H. (2021). Applications of deep learning in fundus images: A review. Med. Image Anal..

[21] Badar M., Haris M., Fatima A. (2020). Application of deep learning for retinal image analysis: A review. Comput. Sci. Rev..

[22] Barros D.M.S., Moura J.C.C., Freire C.R., Taleb A.C., Valentim R.A.M., Morais P.S.G. (2020). Machine learning applied to retinal image processing for glaucoma detection: Review and perspective. Biomed. Eng. Online.

[23] Nuzzi R., Boscia G., Marolo P., Ficardi F. (2021). The Impact of Artificial Intelligence and Deep Learning in Eye Diseases: A Review. Front. Med..

[24] Chen C., Chuah J.H., Ali R., Wang Y. (2021). Retinal Vessel Segmentation Using Deep Learning: A Review. IEEE Access.

[25] Selvam S., Kumar T., Fruttiger M. (2018). Retinal Vasculature development in health and disease. Prog. Retin. Eye Res..

[26] Kumar V., Abbas A., Aster J., Salamat M.S. (2009). Robbins & Cotran Pathologic Basis of Disease.

[27] Microaneurysm. https://www.sciencedirect.com/topics/medicine-and-dentistry/microaneurysm.

[28] Cotton Wool Spots. https://www.sciencedirect.com/topics/medicine-and-dentistry/cotton-wool-spots.

[29] Retinal Hemorrhage. https://www.ncbi.nlm.nih.gov/books/NBK560777.

[30] Esmann V., Lundbæk L., Madsen P.H. (1963). Types of Exudates in Diabetic Retinopathy. J. Intern. Med..

[31] Neely K.A., Gardner T.W. (1998). Ocular Neovascularization. Am. J. Pathol..

[32] Ministry of Health of New Zealand (2016). Diabetic Retinal Screening, Grading, Monitoring and Referral Guidance.

[33] Davis M.D., Gangnon R.E., Lee L.Y., Hubbard L.D., Klein B.E.K., Klein R., Ferris F.L., Bressler S.B., Milton R.C. (2005). The Age-Related Eye Disease Study severity scale for age-related macular degeneration: AREDS Repot No. 17. Arch Ophthalmol..

[34] Seddon J.M., Sharma S., Adelman R.A. (2006). Evaluation of the clinical age-related maculopathy staging system. Ophthalmology.

[35] Thomas R., Loibl K., Parikh R. (2011). Evaluation of a glaucoma patient. Indian J. Ophthalmol..

[36] Weinreb R.N., Aung T., Medeiros F.A. (2014). The Pathophysiology and Treatment of Glaucoma. JAMA.

[37] Dong L., Yang Q., Zhang R.H., Wei W.B. (2021). Artificial intelligence for the detection of age-related macular degeneration in color fundus photographs: A systematic review and meta-analysis. EclinicalMedicine.

[38] Mookiah M.R.K., Hogg S., MacGillivray T.J., Prathiba V., Pradeepa R., Mohan V., Anjana R.M., Doney A.S., Palmer C.N.A., Trucco E. (2021). A review of machine learning methods for retinal blood vessel segmentation and artery/vein classification. Med. Image Anal..

[39] Alyoubi W.L., Shalash W.M., Abulkhair M.F. (2020). Diabetic retinopathy detection through deep learning techniques: A review. Inform. Med. Unlocked.

[40] Thompson A.C., Jammal A.A., Medeiros F.A. (2020). A Review of Deep Learning for Screening, Diagnosis, and Detection of Glaucoma Progression. Transl. Vis. Sci. Technol..

[41] Neuroanatomy, Retina. https://www.ncbi.nlm.nih.gov/books/NBK545310.

[42] Maji D., Santara A., Ghosh S., Sheet D., Mitra P. Deep neural network and random forest hybrid architecture for learning to detect retinal vessels in fundus images. Proceedings of the 37th Annual International Conference of the IEEE Engineering in Medicine and Biology Society (EMBC).

[43] Wang S., Yin Y., Cao G., Wei B., Zheng Y., Yang G. (2015). Hierarchical retinal blood vessel segmentation based on feature and ensemble learning. Neurocomputing.

[44] Fu H., Xu Y., Wong D.W.K., Liu J. Retinal vessel segmentation via deep learning network and fully-connected conditional random fields. Proceedings of the 13th International Symposium on Biomedical Imaging (ISBI).

[45] Fu H., Xu Y., Lin S., Wong D.W.K., Liu J. DeepVessel: Retinal Vessel Segmentation via Deep Learning and Conditional Random Field. Proceedings of the 19th International Conference on Medical Image Computing and Computer-Assisted Intervention (MICCAI).

[46] Luo Y., Yang L., Wang L., Cheng H. Efficient CNN-CRF Network for Retinal Image Segmentation. Proceedings of the 3rd International Conference on Cognitive Systems and Signal Processing (ICCSIP).

[47] Dasgupta A., Singh S. A fully convolutional neural network based structured prediction approach towards the retinal vessel segmentation. Proceedings of the 14th International Symposium on Biomedical Imaging (ISBI).

[48] Meyer M.I., Costa P., Galdran A., Mendonça A.M., Campilho A. A Deep Neural Network for Vessel Segmentation of Scanning Laser Ophthalmoscopy Images. Proceedings of the 14th International Conference Image Analysis and Recognition (ICIAR).

[49] Hu K., Zhang Z., Niu X., Zhang Y., Cao C., Xiao F., Gao X. (2018). Retinal vessel segmentation of color fundus images using multiscale convolutional neural network with an improved cross-entropy loss function. Neurocomputing.

[50] Yan Z., Yang X., Cheng K. (2019). A Three-Stage Deep Learning Model for Accurate Retinal Vessel Segmentation. IEEE J. Biomed. Health Inform..

[51] Park K., Choi S.H., Lee J.Y. (2020). M-GAN: Retinal Blood Vessel Segmentation by Balancing Losses Through Stacked Deep Fully Convolutional Networks. IEEE Access.

[52] Rizzi A., Gatta C., Marini D. (2003). A new algorithm for unsupervised global and local color correction. Pattern Recognit. Lett..

[53] Mao X., Li Q., Xie H., Lau R.Y.K., Wang Z., Smolley S.P. Least Squares Generative Adversarial Networks. Proceedings of the IEEE International Conference on Computer Vision (ICCV).

[54] Oliveira W.S., Teixeira J.V., Ren T.I., Cavalcanti G.D.C., Sijbers J. (2016). Unsupervised Retinal Vessel Segmentation Using Combined Filters. PLoS ONE.

[55] Srinidhi C.L., Aparna P., Rajan J. (2018). A visual attention guided unsupervised feature learning for robust vessel delineation in retinal images. Biomed. Signal Process. Control.

[56] Chaudhuri S., Chatterjee S., Katz N., Nelson M., GoldBaum M. (1989). Detection of blood vessels in retinal images using two-dimensional matched filters. IEEE Trans. Med. Imaging.

[57] Frangi A.F., Niessen W.J., Vincken K.L., Viergever M.A. Multiscale vessel enhancement filtering. Proceedings of the 1st International Conference on Medical Image Computing and Computer-Assisted Intervention (MICCAI).

[58] Soares J.V.B., Leandro J.J.G., Cesar R.M., Jelinek H.F., Cree M.J. (2006). Retinal vessel segmentation using the 2-D Gabor wavelet and supervised classification. IEEE Trans. Med. Imaging.

[59] Goldberg D.E. (1989). Genetic Algorithms in Search Optimization and Machine Learning.

[60] Dunn J.C. (1973). A Fuzzy Relative of the ISODATA Process and Its Use in Detecting Compact Well-Separated Clusters. Cybern. Syst..

[61] Li C., Kao C., Gore J.C., Ding Z. (2008). Minimization of Region-Scalable Fitting Energy for Image Segmentation. IEEE Trans. Image Process..

[62] Joshi G.D., Sivaswamy J. Colour Retinal Image Enhancement Based on Domain Knowledge. Proceedings of the 6th Indian Conference on Computer Vision, Graphics & Image Processing (ICVGIP).

[63] Coates A., Ng A.Y., Montavon G., Orr G.B., Müller K. (2012). Learning Feature Representation with K-Means. Neural Networks: Tricks of the Trade.

[64] Coates A., Ng A., Lee H. An Analysis of Single-Layer Networks in Unsupervised Feature Learning. Proceedings of the 14th International Conference on Artificial Intelligence and Statistics Conference (AISTATS).

[65] Welikala R.A., Foster P.J., Whincup P.H., Rudnicka A.R., Owen C.G., Strachan D.P., Barman S.A. (2017). Automated arteriole and venule classification using deep learning for retinal images from the UK Biobank cohort. Comput. Biol. Mod..

[66] Girard F., Kavalec C., Cheriet F. (2019). Joint segmentation and classification of retinal arteries/veins from fundus images. Artif. Intell. Med..

[67] Yang J., Dong X., Hu Y., Peng Q., Tao G., Ou Y., Cai H., Yang X. (2020). Fully Automatic Arteriovenous Segmentation in Retinal Images via Topology-Aware Generative Adversarial Networks. Interdiscip. Sci..

[68] Mirsharif Q., Tajeripour F., Pourreza H. (2013). Automated characterization of blood vessels as arteries and veins in retinal images. Comput. Med. Imaging. Graph..

[69] Hu Q., Abràmoff M., Garvin M.K. (2015). Automated construction of arterial and venous trees in retinal images. J. Med. Imaging.

[70] Xu X., Ding W., Abràmoff M.D., Cao R. (2017). An improved arteriovenous classification method for the early diagnostics of various diseases in retinal image. Comput. Methods Programs Biomed..

[71] Huang F., Dashtbozorg B., Romeny B.M.H. (2018). Artery/vein classification using reflection features in retina fundus images. Mach. Vis. Appl..

[72] Vijayakumar V., Koozekanani D.D., White R., Kohler J., Roychowdhury S., Parhi K.K. Artery/vein classification of retinal blood vessels using feature selection. Proceedings of the 38th Annual International Conference of the IEEE Engineering in Medicine and Biology Society (EMBC).

[73] Zhao Y., Xie J., Zhang H., Zheng Y., Zhao Y., Qi H., Zhao Y., Su P., Liu J., Liu Y. (2020). Retinal Vascular Network Topology Reconstruction and Artery/Vein Classification via Dominant Set Clustering. IEEE Trans. Med. Imaging.

[74] Srinidhi C.L., Aparna P., Rajan J. (2019). Automated Method for Retinal Artery/Vein Separation via Graph Search Metaheuristic Approach. IEEE Trans. Image Process..

[75] Cheng J., Liu J., Xu Y., Yin F., Wong D.W.K., Tan N., Tao D., Cheng C., Aung T., Wong T.Y. (2013). Superpixel Classification Based Optic Disc and Optic Cup Segmentation for Glaucoma Screening. IEEE Trans. Med. Imaging.

[76] Zhao Y., Rada L., Chen K., Harding S.P., Zheng Y. (2015). Automated Vessel Segmentation Using Infinite Perimeter Active Contour Model with Hybrid Region Information with Application to Retinal Images. IEEE Trans. Med. Imaging.

[77] Pavan M., Pelillo M. (2007). Dominant sets and pairwise clustering. IEEE Trans. Pattern Anal. Mach. Intell..

[78] Srinidhi C.L., Rath P., Sivaswamy J. A Vessel Keypoint Detector for junction classification. Proceedings of the IEEE 14th International Symposium on Biomedical Imaging (ISBI).

[79] Foracchia M., Grisan E., Ruggeri A. (2005). Luminosity and contrast normalization in retinal images. Med. Image Anal..

[80] When Glaucomatous Damage Isn’t Glaucoma. https://www.reviewofophthalmology.com/article/when-glaucomatous-damage-isnt-glaucoma.

[81] When It’s Not Glaucoma. https://www.aao.org/eyenet/article/when-its-not-glaucoma.

[82] Leung C.K., Cheung C.Y., Weinreb R.N., Qui Q., Liu S., Li H., Xu G., Fan N., Huang L., Pang C. (2009). Retinal nerve fiber layer imaging with spectral-domain optical coherence tomography: A variability and diagnostic performance study. Ophthalmology.

[83] Szegedy C., Ioffe S., Vanhoucke V., Alemi A.A. Inception-v4, inception-ResNet and the impact of residual connections on learning. Proceedings of the 31st AAAI Conference on Artificial Intelligence.

[84] Simonyan K., Zisserman A. Very Deep Convolutional Networks for Large-Scale Image Recognition. Proceedings of the 3rd International Conference on Learning Representations (ICLR).

[85] Szegedy C., Vanhoucke V., Ioffe S., Shlens J., Wojna Z. Rethinking the Inception Architecture for Computer Vision. Proceedings of the IEEE Conference on Computer Vision and Pattern Recognition (CVPR).

[86] Howard A.G., Zhu M., Chen B., Kalenichenko D. (2017). MobileNets: Effcient Convolutional Neural Networks for Mobile Vision Applications. arXiv.

[87] Iandola F.N., Han S., Moskewicz M.W., Ahshraf K., Dally W.J., Keutzer K. (2016). SqueezeNet: AlexNet-level accuracy with 50x fewer parameters and <0.5MB model size. arXiv.

[88] Chollet F. Xception: Deep Learning with Depthwise Separablee Convolutions. Proceedings of the IEEE Conference on Computer Vision and Pattern Recognition (CVPR).

[89] Huang G., Liu Z., Maaten L.V., Weinberger K.Q. Densely Connected Convolutional Networks. Proceedings of the IEEE Conference on Computer Vision and Pattern Recognition (CVPR).

[90] Krizhevsky A., Sutskever I., Hinton G.E. ImageNet Classification with Deep Convolutional Neural Networks. Proceedings of the 26th Annual Conference on Neural Information Processing Systems (NIPS).

[91] Szegedy C., Liu W., Jia Y., Sermanet P., Reed S., Anguelov D., Erhan D., Vanhoucke V., Rabinovich A. Going deeper with convolutions. Proceedings of the IEEE Conference on Computer Vision and Pattern Recognition (CVPR).

[92] Jiang J., Yang K., Gao M., Zhang D., Ma H., Qian W. An Interpretable Ensemble Deep Learning Model for Diabetic Retinopathy Disease Classification. Proceedings of the 41th Annual International Conference of the IEEE Engineering in Medicine and Biology Society (EMBC).

[93] Dutta S., Manideep B.C., Basha S.M., Caytiles R.D., Iyengar N. (2018). Classification of Diabetic Retinopathy Images by Using Deep Learning Models. Int. J. Grid Dist. Comput..

[94] Pires R., Avila S., Wainer J., Valle E., Abramoff M.D., Rocha A. (2019). A data-driven approach to referable diabetic retinopathy detection. Artif. Intell. Med..

[95] Zhang W., Zhong J., Yang S., Gao Z., Hu J., Chen Y., Yi Z. (2019). Automated identification and grading system of diabetic retinopathy using deep neural network. Knowl. Based. Syst..

[96] Rehman M., Khan S.H., Abbas Z., Rizvi S.M.D. Classification of Diabetic Retinopathy Images Based on Customised CNN Architecture. Proceedings of the Amity International Conference on Artificial Intelligence (AICAI).

[97] Wang X., Lu Y., Wang Y., Chen W. Diabetic Retinopathy Stage Classification Using Convolutional Neural Networks. Proceedings of the IEEE International Conference on Information Reuse and Integration (IRI).

[98] Oh K., Kang H.M., Leem D., Lee H., Seo K.Y., Yoon S. (2021). Early detection of diabetic retinopathy based on deep learning and ultra-wide-field fundus images. Sci. Rep..

[99] Adem K. (2018). Exudate detection for diabetic retinopathy with circular Hough transformation and convolutional neural networks. Expert Syst. Appl..

[100] Wang H., Yuan G., Zhao X., Peng L., Wang Z., He Y., Qu C., Peng Z. (2020). Hard exudate detection based on deep model learned information and multi-feature joint representation for diabetic retinopathy screening. Comput. Methods Programs Biomed..

[101] Chudzik P., Majumdar S., Calivá F., Al-Diri B., Hunter A. (2018). Microaneurysm detection using fully convolutional neural networks. Comput. Methods Programs Biomed..

[102] Grinsven M.J.J.P., Ginneken B., Hoyng C.B., Theelen T., Sánchez C.I. (2020). Fast Convolutional Neural Network Training Using Selective Data Sampling: Application to Hemorrhage Detection in Color Fundus Images. IEEE Trans. Med. Imaging.

[103] Zago G.T., Andreão R.V., Dorizzi B., Salles E.O.T. (2020). Diabetic retinopathy detection using red lesion localization and convolutional neural networks. Comput. Biol. Med..

[104] Alyoubi W.L., Abulkhair M.F., Shalash W.M. (2021). Diabetic Retinopathy Fundus Image Classification and Lesions Localization System Using Deep Learning. Sensors.

[105] Grassmann F., Mengelkamp J., Brandl C., Harsch S., Zimmermann M.E., Linkohr B., Peters A., Heid I.M., Palm C., Weber B.H. (2018). A Deep Learning Algorithm for Prediction of Age-related Eye Disease Study Severity Scale for Age-related Macular Degeneration from Color Fundus Photography. Ophthalmology.

[106] Bhuiyan A., Wong T.Y., Ting D.S.W., Govindaiah A., Souied E.H., Smith T.R. (2020). Artificial Intelligence to Stratify Severity of Age-Related Macular Degenration (AMD) and Predict Risk of Progression to Late AMD. Transl. Vis. Sci. Technol..

[107] Li F., Yan L., Wang Y., Shi J., Chen H., Zhang X., Jiang M., Wu Z., Zhou K. (2020). Deep learning-based automated detection of glaucomatous optic neuropathy on color fundus photographs. Graefes. Arch. Clin. Exp. Ophthalmol..

[108] Redmon J., Farhadi A. (2018). YOLOv3: An Incremental Improvement. arXiv.

[109] Tan M., Le Q. EfficientNet: Rethinking Model Scaling for Convolutional Neural Networks. Proceedings of the 36th International Conference on Machine Learning (ICML).

[110] Burlina P.M., Joshi N., Pekala M., Pacheco K.D., Freund D.E., Bressler N.M. (2017). Automated Grading of Age-Related Macular Degeneration From Color Fundus Images Using Deep Convolutional Neural Networks. JAMA Ophthalmol..

[111] González-Gonzalo C., Sánchez-Gutiérrez V., Hernández-Martínez P., Contreras I., Lechanteur Y.T., Domanian A., Ginneken B., Sánchez C. (2020). Evaluation of a deep learning system for the joint automated detection of diabetic retinopathy and age-related macular degeneration. Acta Ophthalmol..

[112] Keel S., Li Z., Scheetz J., Robman L., Phung J., Makeyeva G., Aung K., Liu C., Yan X., Meng W. (2019). Development and validation of a deep-learning algorithm for the detection of a deep-learning algorithm for the detection of neovascular age-related macular degeneration from colour fundus photographs. Clin. Exp. Ophthalmol..

[113] Sermanet P., Eigen D., Zhang X., Mathieu M., Fergus R., LeCun Y. (2014). OverFeat: Integrated Recognition, Localization and Detection using Convolutional Networks. arXiv.

[114] Burlina P., Pacheeco K.D., Joshi N., Freund D.E., Bressler N.M. (2017). Comparing humans and deep learning performance for grading AMD: A study in using universal deep features and transfer learning for automated AMD analysis. Comput. Biol. Med..

[115] Peng Y., Dharssi S., Chen Q., Keenan T.D., Agrón E., Wong W.T., Chew E.Y., Lu Z. (2019). DeepSeeNet: A Deep Learning Model for Automated Classification of Patient-based Age-related Macular Degeneration Severity from Color Fundus Photographs. Ophthalmology.

[116] Keenan T.D., Dharssi S., Peng Y., Wong W.T., Lu Z., Chew E.Y. (2019). A Deep Learning Approach for Automated Detection of Geographic Atrophy from Color Fundus Photographs. Ophthalmology.

[117] Hussain M.A., Govindaiah A., Souied E., Smith R.T., Bhuiyan A. Automated tracking and change detection for Age-related Macular Degeneration Progression using retinal fundus imaging. Proceedings of the 7th International Conference on Informatics, Electronics & amp (ICIEV).

[118] Zoph B., Vasudevan V., Shlens J., Le Q.V. Learning Transferable Architectures for Scalable Image Recognition. Proceedings of the IEEE/CVF Conference on Computer Vision and Pattern Recognition (CVPR).

[119] Prum B.E., Rosenberg L.F., Gedde S.J., Mansberger S.L., Stein J.D., Moroi S.E., Herndon L.W., Lim M.C., Williams R.D. (2016). Primary Open-Angle Glaucoma Preferred Practice Pattern(^®^) Guidelines. Ophthalmology.

[120] Hollands H., Johnson D., Hollands S., Simel D.L., Jinapriya D., Sharma S. (2013). Do findings on routine examination identify patients at risk for primary open-angle glaucoma? The rational clinical examination systematic review. JAMA.

[121] Fingeret M., Medeiors F.A., Susanna R., Weinreb R.N. (2005). Five rules to evaluate the optic disc and retinal nerve fiber layer for glaucoma. Optometry.

[122] Shinde R. (2021). Glaucoma detection in retinal fundus images using U-Net and supervised machine learning algorithms. Intell. Based Med..

[123] Singh L.K., Garg H., Khanna M., Bhadoria R.H. (2021). An enhanced deep image model for glaucoma diagnosis using feature-based detection in retinal fundus. Med. Biol. Eng. Comput..

[124] Li Z., He Y., Keel S., Meng W., Chang R.T., He M. (2018). Efficacy of a Deep Learning System for Detecting Glaucomatous Optic Neuropathy Based on Color Fundus Photographs. Ophthalmology.

[125] The Japan Glaucoma Society Guidelines for Glaucoma (4th Edition). http://journal.nichigan.or.jp/Search?chk0=on&searchfull2=The+Japan+Glaucoma+Society+Guidelines+for+Glaucoma.

[126] Shibata N., Masaki T., Mitsuhashi K., Fujino Y., Matsuura M., Murata H., Asaoka R. (2018). Development of a deep residual learning algorithm to screen for glaucoma from fundus photography. Sci. Rep..

[127] Kim M., Park H., Zuallaert J., Janssens O., Hoeecke S., Neve W. Computer-Aided Diagnosis and Localization of Glaucoma Using Deep Learning. Proceedings of the IEEE International Conference on Bioinformatics and Biomedicine (BIBM).

[128] Phene S., Dunn R.C., Hammel N., Liu Y., Krause J., Kitade N., Schaekermann M., Sayres R., Wu D.J., Bora A. (2019). Deep Learning and Glaucoma Specialists. Ophthalmology.

[129] Deng Z., Li Y., Wang Y., Yue Q., Yang Z., Boumediene D., Carloganu C., Français V., Cho G., Kim D.-W. (2017). Tracking within Hadronic Showers in the CALICE SDHCAL prototype using a Hough Transform Technique. J. Instrum..

[130] Li T., Gao Y., Wang K., Guo S., Liu H., Kang H. (2019). Diagnostic assessment of deep learning algorithms for diabetic retinopathy screening. Inf. Sci..

[131] Shanti T., Sabeenian R. (2019). Modified Alexnet architecture for classification of diabetic for classification of diabetic retinopathy images. Comput. Electr. Eng..

[132] Zapata M., Royo-Fibla D., Font O., Vela J., Marcantonio I., Moya-Sanchez E., Sanchez-Perez A., Garcia-Gasulla D., Cortes U., Ayguade E. (2020). Artificial intelligence to identify retinal fundus images, quality validation, laterality evaluation, macular degeneration, and suspected glaucoma. Clin. Ophthalmol..

[133] Liu H., Li L., Wormstone M., Qiao C., Zhang C., Liu P., Li S., Wang H., Mou D., Pang R. (2019). Development and Validation of a Deep Learning System to Detect Glaucomatous Optic Neuropathy Using Fundus Photographs. JAMA Ophthalmol..

[134] Christopher M., Belghith A., Bowd C., Proudfoot J.A., Goldbaum M.H., Weinreb R.N., Girkin C.A., Liebmann J.M., Zangwill L.M. (2018). Performance of Deep Learning Architectures and Transfer Learning for Detecting Glaucomatous Optic Neuropathy in Fundus Photographs. Sci. Rep..

[135] Zhou B., Khosla A., Lapedriza A., Oliva A., Torralba A. Learning Deep Features for Discriminative Localization. Proceedings of the IEEE Conference on Computer Vision and Pattern Recognition (CVPR).

[136] Selvaraju R., Cogswell M., Das A., Vedantam R., Parika D., Batra D. Grad-CAM: Visual Explanations from Deep Networks via Gradient-based Localization. Proceedings of the IEEE International Conference on Computer Vision (ICCV).

[137] Anowar F., Sadaoui S., Selim B. (2021). Conceptual and empirical comparison of dimensionality reduction algorithms (PCA, KPCA, LDA, MDS, SVD, LLE, ISOMAP, LE, ICA, t-SNE). Comput. Sci. Rev..

[138] Sisson C.P., Farnand S., Fairchild M., Fischer B. (2014). Analysis of Color Consistency in Retinal Fundus Photography: Application of Color Management and Development of an Eye Model Standard. Anal. Cell. Pathol..

[139] Jampel H.D., Friedman D., Quigley H., Vitale S., Miller R., Knezevich F., Ding Y. (2009). Agreement among glaucoma specialists in assessing progressive disc changes from photographs in open-angle glaucoma patients. Am. J. Ophthalmol..

[140] Abrams L.S., Scott I.U., Spaeth G.L., Quigley H.A., Varma R. (1994). Agreement among optometrists, ophthalmologists, and residents in evaluating the optic disc for glaucoma. Ophthalmology.

[141] Tatham A.J., Medeiros F.A. (2017). Detecting Structural Progression in Glaucoma with Optical Coherence Tomography. Ophthalmology.

[142] Medeiros F.A., Jammal A.A., Thompson A.C. (2019). From Machine to Machine: An OCT-Trained Deep Learning Algorithm for Objective Quantification of Glaucomatous Damage in Fundus Photographs. Ophthalmology.

[143] Reznicek L., Burzer S., Laubichler A., Nasseri A., Lohmann C.P., Feucht N., Ulbig M., Maier M. (2017). Structure-function relationship comparison between retinal nerve fibre layer and Bruch’s membrane opening-minimum rim width in glaucoma. Int. J. Ophthalmol..

[144] Thompson A., Jammal A.A., Medeiors F.A. (2019). A Deep Learning Algorithm to Quantify Neuroretinal Rim Loss from Optic Disc Photographs. Am. J. Ophthalmol..

